# N-Terminomics TAILS Identifies Host Cell Substrates of Poliovirus and Coxsackievirus B3 3C Proteinases That Modulate Virus Infection

**DOI:** 10.1128/JVI.02211-17

**Published:** 2018-03-28

**Authors:** Julienne M. Jagdeo, Antoine Dufour, Theo Klein, Nestor Solis, Oded Kleifeld, Jayachandran Kizhakkedathu, Honglin Luo, Christopher M. Overall, Eric Jan

**Affiliations:** a Department of Biochemistry and Molecular Biology, University of British Columbia, Vancouver, British Columbia, Canada; b Life Sciences Institute, University of British Columbia, Vancouver, British Columbia, Canada; c Centre for Blood Research, Faculty of Dentistry, Department of Oral Biological and Medical Sciences, University of British Columbia, Vancouver, British Columbia, Canada; d Department of Pathology and Laboratory Medicine, University of British Columbia, Vancouver, British Columbia, Canada; e School of Biomedical Sciences, Monash University, Victoria, Australia; University of Texas Southwestern Medical Center

**Keywords:** RNA replication, coxsackievirus, enterovirus, plus-strand RNA virus, poliovirus, proteases, proteinase, proteomics

## Abstract

Enteroviruses encode proteinases that are essential for processing of the translated viral polyprotein. In addition, viral proteinases also target host proteins to manipulate cellular processes and evade innate antiviral responses to promote replication and infection. Although some host protein substrates of enterovirus proteinases have been identified, the full repertoire of targets remains unknown. We used a novel quantitative *in vitro* proteomics-based approach, termed terminal amine isotopic labeling of substrates (TAILS), to identify with high confidence 72 and 34 new host protein targets of poliovirus and coxsackievirus B3 (CVB3) 3C proteinases (3C^pro^s) in HeLa cell and cardiomyocyte HL-1 cell lysates, respectively. We validated a subset of candidate substrates that are targets of poliovirus 3C^pro^ *in vitro* including three common protein targets, phosphoribosylformylglycinamidine synthetase (PFAS), hnRNP K, and hnRNP M, of both proteinases. 3C^pro^-targeted substrates were also cleaved in virus-infected cells but not noncleavable mutant proteins designed from the TAILS-identified cleavage sites. Knockdown of TAILS-identified target proteins modulated infection both negatively and positively, suggesting that cleavage by 3C^pro^ promotes infection. Indeed, expression of a cleavage-resistant mutant form of the endoplasmic reticulum (ER)-Golgi vesicle-tethering protein p115 decreased viral replication and yield. As the first comprehensive study to identify and validate functional enterovirus 3C^pro^ substrates *in vivo*, we conclude that N-terminomics by TAILS is an effective strategy to identify host targets of viral proteinases in a nonbiased manner.

**IMPORTANCE** Enteroviruses are positive-strand RNA viruses that encode proteases that cleave the viral polyprotein into the individual mature viral proteins. In addition, viral proteases target host proteins in order to modulate cellular pathways and block antiviral responses in order to facilitate virus infection. Although several host protein targets have been identified, the entire list of proteins that are targeted is not known. In this study, we used a novel unbiased proteomics approach to identify ∼100 novel host targets of the enterovirus 3C protease, thus providing further insights into the network of cellular pathways that are modulated to promote virus infection.

## INTRODUCTION

Proteases regulate numerous cellular processes through cleavage of their substrates (1). Several classes of viruses encode proteinases that function in processing and maturation of viral proteins for the synthesis of new virus particles. In addition, viral proteinases can target a subset of host proteins to modulate cellular processes that facilitate virus infection (2). Viral proteinases have been well characterized among the picornavirus family, which includes members of the Enterovirus genus, poliovirus (PV) and coxsackievirus B3 (CVB3). Enterovirus infections can cause a wide range of diseases, from respiratory ailments to paralysis and dilated cardiomyopathy, for which there are no effective antiviral therapies (3–5).

Picornaviruses possess a positive-sense single-stranded RNA genome approximately ∼7.5 kb in length that contains a single open reading frame (6–8). A highly structured internal ribosome entry site (IRES) within the 5′ untranslated region (UTR) directs viral translation to produce a single polyprotein, which is then processed into individual mature viral proteins by at least one virally encoded proteinase. During infection, processing of the viral polyprotein occurs through a coordinated sequence of cleavage events in a site-specific and temporally regulated manner (9). The 3C proteinase (3C^pro^), a chymotrypsin-like protease with a cysteine nucleophile, is conserved among all known picornaviruses (10, 11). 3C^pro^ in its precursor form as 3CD^pro^ is responsible for the majority of the viral polyprotein cleavages, targeting distinct glutamine-glycine residues with a preferred consensus cleavage motif of AXXQ↓GPXX, where X denotes any amino acid and the down arrow represents the scissile bond between the P1′ to P4′ and P1 to P4 residues, respectively (12). The Enterovirus genus of picornaviruses encodes a second proteinase, the 2A proteinase (2A^pro^), that performs minor cleavage events within the polyprotein. Similar to 3C^pro^, 2A^pro^ bears a chymotrypsin-like structure with a cysteine nucleophile and mediates a single cleavage event within the polyprotein at its N terminus between specific tyrosine-glycine residues (13).

The identification of several host targets of picornaviral proteinases has provided insights into the fundamental virus-host interactions and the viral strategies utilized to modulate and usurp host processes to facilitate specific steps of the viral life cycle. The classic example is cleavage of the translation initiation factor, eukaryotic initiation factor 4G (eIF4G), by 2A^pro^, which contributes to the shutoff of host translation, a prominent characteristic of many picornavirus infections that serves to inhibit induction of host antiviral responses and to favor viral IRES-mediated translation (14–16). Enterovirus proteinases also target proteins involved in transcription, nuclear import, RNA metabolism, and antiviral innate immune response signaling (17, 18). The functions of many of these host proteins are hijacked to support various steps in the life cycle, which can be regulated through cleavage. For example, in poliovirus-infected cells, relocalized poly(rC) binding protein 2 (PCBP2; also called hnRNP E2) and polypyrimidine tract binding protein (PTB; also called hnRNP I) bind to distinct regions within the viral 5′ UTR to promote viral translation (19–22). As infection progresses, a switch from viral translation to replication occurs whereby PCBP2 and PTB are cleaved by 3C^pro^, thus disrupting their ability to facilitate virus translation.

Currently, there are ∼54 known host targets of picornavirus proteinases (17). Most targets have been identified through candidate approaches, two-dimensional (2D) gel electrophoresis coupled with mass spectrometry (MS), and bioinformatics based on a search for consensus cleavage sites (21, 23–26). However, these techniques have several limitations and biases (27). Bioinformatics and candidate approaches are hypothesis driven and may not fully capture physiologically relevant protease substrates, and 2D gel electrophoresis followed by mass spectrometry is older technology that has limited resolution and coverage and may miss low-abundance target proteins. To overcome these limitations, recent advances in mass spectrometry-based techniques have developed gel-free strategies that identify protease-generated peptides (28–30). One such approach dedicated for the enrichment of protease cleavage products is terminal amine isotopic labeling of substrates (TAILS). Protease-generated N terminus (neo-N terminus) peptides are purified by negative selection and then identified by tandem mass spectrometry (MS/MS) (30). The advantage of TAILS is that minimal amounts of lysate are required, and identification of the cleaved peptide by mass spectrometry analysis simultaneously identifies the protein and the actual cleavage site. TAILS has successfully identified novel substrates for several cellular proteases (31–34). Here, we applied TAILS to identify novel substrates of the picornavirus proteinase 3C^pro^ from the enteroviruses poliovirus and CVB3. We identified multiple high-confidence candidate substrates of poliovirus and CVB3 3C^pro^ and validated several *in vitro* and in infection. Three candidate substrates were found in common, which were identified at the same cleavage site, suggesting that they may be strategic targets for enterovirus infection. Moreover, small interfering RNA (siRNA)-mediated depletion studies revealed functional significance of candidate targets in promoting poliovirus infection.

## RESULTS

### TAILS identification of candidate substrates of poliovirus and CVB3 3C^pro^ *in vitro*.

**FIG 1:**
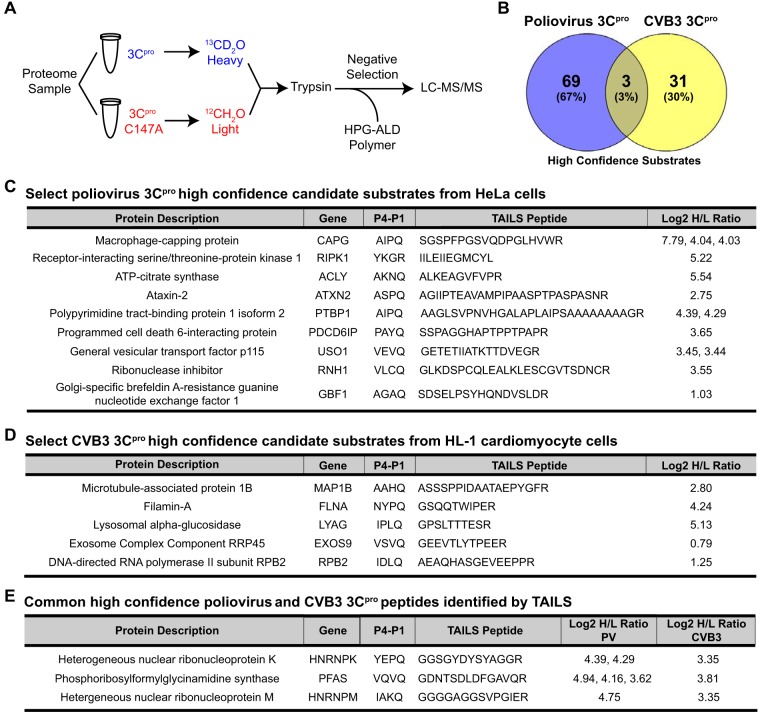
High-confidence candidate substrates of poliovirus and CVB3 3C^pro^ identified by TAILS. (A) TAILS 3C^pro^ workflow. Proteome sample (500 μg) extracted from HeLa or HL-1 cell extracts was incubated with poliovirus 3C^pro^ or CVB3 3C^pro^ (100 ng/μl), respectively, followed by isotopic dimethylation labeling and TAILS. High-confidence substrates were determined by box plot-and-whiskers analysis of the quantified heavy/light (H/L) ratio of dimethylation-labeled semitryptic neo-N terminus peptides. HPG-ALD, hyperbranched polyglycerol-aldehyde. (B) Venn diagram illustrating the percentage of common high-confidence substrates identified between TAILS analysis of poliovirus and CVB3 3C^pro^. (C and D) Select high-confidence substrates of poliovirus 3C^pro^ from HeLa cell extracts and of CVB3 3C^pro^ from HL-1 cardiomyocytes. (E) Common peptides identified among both the poliovirus and CVB3 3C^pro^ list of high-confidence substrates. Peptides listed are statistically significant high-H/L semitryptic neo-N terminus peptides. All high-confidence substrates are listed in Tables S5 and S9 in the supplemental material.

Although ∼40 substrates of picornavirus 3C^pro^ are known, we hypothesized that 3C^pro^ targets other host substrates that have yet to be identified (17). To address this, we applied an *in vitro* TAILS approach using proteinases from two model enteroviruses, poliovirus and CVB3 3C^pro^ (Fig. 1A). An *in vitro* approach was chosen in order to identify direct host targets of the viral proteinases and is a simple approach that has been used successfully to identify bona fide substrates (29, 30, 35, 36). Poliovirus and CVB3 3C proteinases were chosen as they have been extensively studied at the biochemical and structural levels, and thus conditions for cleavage are known (11, 37, 38). Moreover, as a subset of host targets are known, they can be used to benchmark the success of TAILS. Briefly, proteome samples were prepared from HeLa cell extracts and incubated with a purified wild-type or a catalytically inactive mutant (Cys147Ala, here referred to as C147A) recombinant poliovirus 3C^pro^. For comparison, proteome samples from HL-1 cardiomyocyte lysates were incubated with a CVB3 wild-type or catalytically inactive (C147A) recombinant 3C^pro^ in order to more closely recapitulate a physiological setting of CVB3 infection (4, 5). CVB3 is a clinically relevant virus that contributes to viral myocarditis and dilated cardiomyopathy (39). TAILS analysis of proteinases from two different enteroviruses that infect distinct cell lines may identify common and tissue-specific host targets that may be important for general enterovirus infection, including those that may contribute to specific pathogenesis of disease.

**FIG 2:**
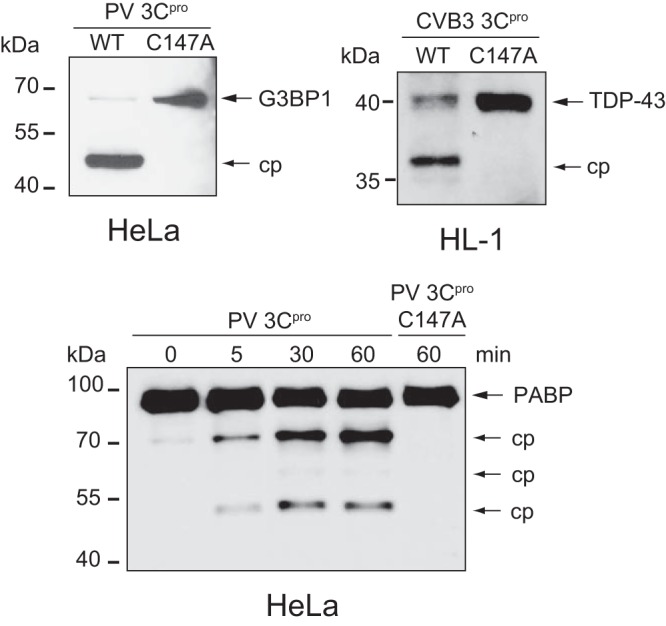
*In vitro* cleavage assay of known enterovirus 3C^pro^ substrates. Immunoblot showing cleavage of G3BP1 (top left) and PABP (bottom) following an *in vitro* cleavage assay with HeLa cell lysates incubated with wild-type (WT) or C147A mutant poliovirus (PV) 3C^pro^ at the times indicated. Immunoblotting was performed following cleavage of TDP-43 (top right) in HL-1 cell lysates incubated with wild-type or C147A mutant coxsackievirus (CVB3) 3C^pro^. cp, cleavage product.

Incubation of wild-type but not mutant 3C^pro^ in lysates resulted in cleavage of the known target substrates poly(A) binding protein (PABP), Ras GTPase-activating protein-binding protein 1 (G3BP1), and TAR DNA-binding protein 43 (TDP-43), thus confirming that the purified recombinant 3C^pro^ is active (Fig. 2) (40–44). TAILS was performed on cellular extracts under conditions previously optimized for proteolytic activity (Fig. 2) (29, 30, 45). Following proteinase digestion, samples were isotopically labeled by reductive dimethylation of primary amines, applying an isotopically heavy (+6 Da) or medium (+4 Da) (defined as H) formaldehyde to the wild-type sample versus a light (L) formaldehyde to the catalytically inactive (C147A) 3C^pro^ mutant sample (Fig. 1A). The samples were mixed and trypsinized, which was followed by a negative-selection step using a dendritic aldehyde polymer that removes trypsin-generated N-terminally unlabeled peptides, thus enriching for neo-N terminus and natural N terminus peptides that were then identified by liquid chromatography tandem mass spectrometry (LC-MS/MS).

For poliovirus 3C^pro^, TAILS-enriched peptides from HeLa cell lysates were analyzed from a total of seven biological replicates using three different mass spectrometers and two search engines for peptide identification (Table 1; see also Tables S1 to S4 in the supplemental material). From the seven biological replicates, we identified 3,482 total peptides, of which 3,210 were unique peptides from 1,965 unique proteins. For CVB3 3C^pro^, using three biological replicates, 347 unique peptides from 240 unique proteins were identified from a total of 364 identified peptides (Tables 1 and S8).

**TABLE 1:** Summary of total peptide and proteins identified by TAILS analysis of poliovirus and CVB3 3C^pro^

3C^pro^ and cell type	Instrument	No. of expts	Total no. of peptides	No. of unique peptides	No. of unique proteins	No. of high-confidence substrates
PV 3C^pro^ in HeLa cell lysates						
	OrbiXL	3	627	534	444	10
	QExactive	2	1,778	1,741	1,059	37
	QToF	2	1,077	759	614	10
	Orbi + QExactive	5	2,253	2,224	1,239	15
Total for the group (unique)		7	3,482	3,210	1,965	72
CVB 3C^pro^ in HL-1 cell lysates	QExactive	3	364	347	240	34

Peptides with a high heavy/light (H/L) isotopic ratio (protease-treated/control) indicative of viral proteinase-generated cleaved peptides were identified and ranked to represent candidate substrates enriched in lysates containing wild-type 3C^pro^ compared to those containing mutant 3C^pro^ (Tables S5, S6, S9, and S10). A box plot-and-whiskers analysis was applied to determine the high and low statistical isotopic H/L ratio cutoffs. Neo-N terminus peptides with an H/L ratio above the high statistical ratio cutoff, representing peptides located internally within the protein, that contained an arginine at the C terminus (semitryptic) were selected as high-confidence candidate substrates (Tables S5 and S9). For poliovirus 3C^pro^, 72 high-confidence candidate substrates were identified, including a peptide from isoform 2 of PTB, which is a known substrate of 3C^pro^ (Fig. 1C and Table S5) (20). Importantly, the PTB peptide identified by TAILS indicated that cleavage occurs at ^311^AIPQ↓AAGL^318^, which is a previously reported cleavage site, thus providing validation of TAILS for 3C^pro^ (20). Although it was surprising that only one known substrate of enterovirus 3C^pro^ was identified by TAILS, predicted neo-N peptides of some known 3C^pro^ protein targets that would be generated by TAILS were either too short or too long for efficient ionization and fragmentation to allow confident identification by mass spectrometry (Table S12). An additional 78 natural N terminus or neo-N terminus nonsemitryptic peptides were identified with an H/L ratio above the statistical cutoff (Table S6). Furthermore, we identified 165 neo-N terminus and natural N terminus peptides with a low H/L ratio, indicative of degradative loss of this part of the molecule by the proteinase (Table S7).

The same approach for substrate winnowing was applied to identify 34 high-confidence candidate substrates of CVB3 3C^pro^, as well as 10 natural N terminus or nonsemitryptic neo-N terminus peptides and 36 significantly low-ratio (degraded) neo-N terminus and natural N terminus peptides (Fig. 1D; Tables 1 and S9 to S11). Interestingly, three TAILS peptides were found to be common among both poliovirus and CVB3 3C^pro^ lists of high-confidence substrates: hnRNP K, hnRNP M, and phosphoribosylformylglycinamidine synthase (PFAS) (Fig. 1B and E). Gene Ontology (GO) analysis showed that the 3C^pro^ high-confidence candidate protein targets are, in general, broadly distributed across many cellular pathways (e.g., a majority of pathways had only one target substrate) with a slight enrichment in apoptosis and ubiquitin proteasome signaling pathways (Table S13). This result is in line with the idea that 3C^pro^ modulates a number of pathways to facilitate infection (17).

**FIG 3:**
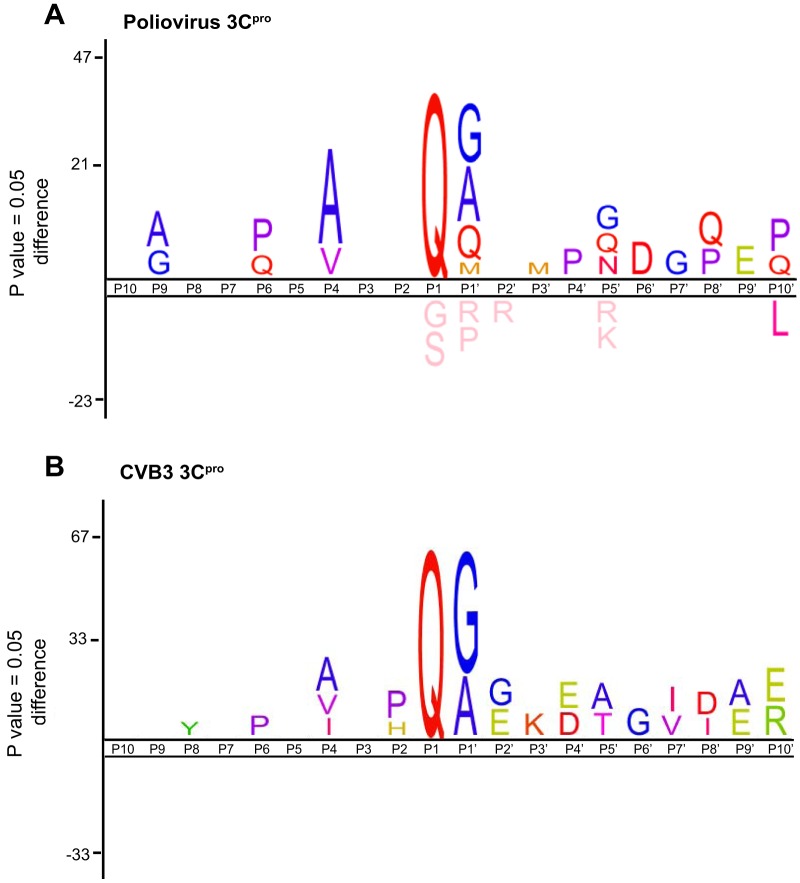
Consensus cleavage site analysis of poliovirus and CVB3 3C^pro^ high-confidence substrate peptides. Sequence logos of the 10 amino acids positioned directly upstream (P1 to P10) and downstream (P1′ to P10′) of the TAILS-identified peptides for poliovirus 3C^pro^ (A) and CVB3 3C^pro^ (B).

An analysis of the poliovirus 3C^pro^ cleavage sites identified by TAILS revealed a strong preference for glutamine and alanine or valine at the P1 and P4 positions, respectively, which is consistent with the consensus cleavage sequence previously derived from mutagenesis studies and peptide substrate analysis (Fig. 3A) (12). In addition, glycine, alanine, and serine were strongly preferred at the P1′ position, similar to what was observed previously (46). However, the additional preference for methionine and glutamine suggests that there is further leniency at the P1′ position. Similarly, analysis of CVB3 3C^pro^ high-confidence substrate peptides shows preferences consistent with the known consensus cleavage sequence, with glutamine at P1 and glycine and alanine at P1′ (Fig. 3B). Alanine was also observed at the P4 positions, along with additional amino acids that may be accommodated in these positions. Additional amino acid preferences were observed for both poliovirus and CVB3 3C^pro^ at other positions where substrate specificity is less well characterized. In summary, TAILS identified several novel candidate substrates of 3C^pro^ and indicated that 3C^pro^ can accommodate a wider range of target cleavage sequences than previously reported.

### Validation of candidate substrates *in vitro*.

**FIG 4:**
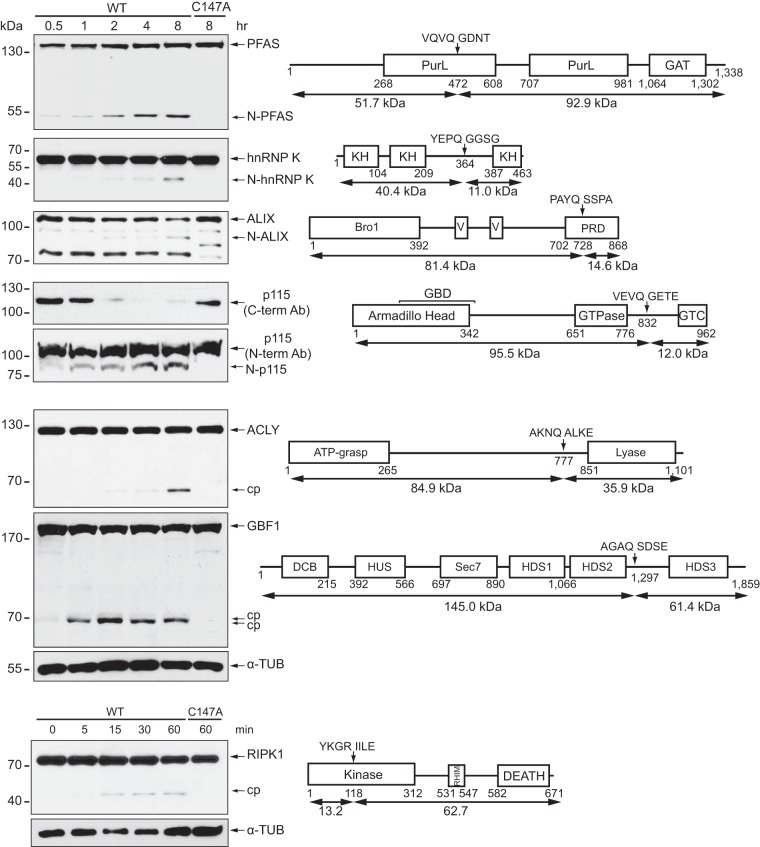
Validation of TAILS high-confidence substrates by *in vitro* cleavage assay. (Left) HeLa cell lysates were incubated with purified wild-type or mutant (C147A) poliovirus 3C^pro^ (100 ng/μl). Proteins were loaded on an SDS-PAGE gel, and cleavage was assessed by immunoblotting. (Right) Schematics of corresponding high-confidence candidate substrates, indicating key domains, the position of TAILS-predicted cleavage sites, and the four amino acid positions directly upstream (P1 to P4) and downstream (P1′ to P4′) of the cleavage site. The predicted molecular masses of the cleavage protein fragments are shown below. cp, cleavage product; N, N-terminal cleavage product; C, C-terminal cleavage product; GAT, glutamine amidotransferase; KH, hnRNP K homology; PRD, proline rich domain; GTC, Golgi tethering complex; HUS, homology upstream of Sec7; HDS, homology downstream of Sec7; RHIM, RIP homotypic interaction motif; DCB, dimerization and cyclophilin binding; α-TUB, α-tubulin; Ab, antibody.

We chose both specific and random candidate substrates for further analysis based on identification in both PV and CVB3 3C^pro^ lists (i.e., hnRNP M and K and PFAS), their potential function in the viral life cycle (i.e., RIPK1 [receptor-interacting serine/threonine-protein kinase 1] in the inflammasome pathway and p115 and Golgi-specific brefeldin A-resistant guanine nucleotide exchange factor 1 [GBF1] in the ER-Golgi transport pathway), and whether putative a TAILS-identified cleavage site had a glutamine at the P1 position and had a statistically significant high heavy/light ratio (e.g., ACLY and ALIX). To confirm whether TAILS-identified candidate substrates are bona fide targets of 3C^pro^, we monitored select substrates by immunoblotting following an *in vitro* cleavage assay. We previously validated and characterized hnRNP M as a novel substrate of poliovirus and CVB3 3C^pro^, which we identified by TAILS of both poliovirus and CVB3 3C^pro^ (Fig. 1E) (45). Addition of recombinant poliovirus wild-type but not mutant 3C^pro^ in HeLa lysates resulted in cleaved PFAS, hnRNP K, programmed cell death 6-interacting protein (ALIX), ATP-citrate synthase (ACLY), GBF1, and RIPK1 (Fig. 4), thereby validating the TAILS data. The masses of the cleavage fragments of PFAS, hnRNP K, ALIX, p115, and GBF1 identified by immunoblotting are consistent with the cleavage sites found by TAILS (Fig. 4). For example, the TAILS-generated peptide of hnRNP K predicts cleavage at ^364^Gln↓Gly^365^ to produce N- and C-terminal protein fragments with masses of ∼40.4 and ∼11.0 kDa, respectively. Immunoblotting using the hnRNP K antibody detected an N-terminal cleavage fragment of ∼40 kDa. Although the general vesicular transport factor p115 antibody that recognizes a C-terminal domain epitope showed a loss of full-length protein in lysates incubated with wild-type 3C^pro^, an N-terminal p115 antibody detected a cleaved N-terminal p115 fragment consistent with the TAILS-identified cleavage site. In contrast, immunoblotting of ACLY and RIPK1 detected different cleavage fragments than those identified by TAILS, suggesting the existence of an alternative cleavage site that could not be identified by MS (e.g., the site was too close to an Arg, rendering it redundant and therefore not considered further bioinformatically) or cleavage by 3C^pro^ at multiple sites. Thus, we have identified seven additional novel protein targets of 3C^pro^, in addition to hnRNP M, through TAILS.

**FIG 5:**
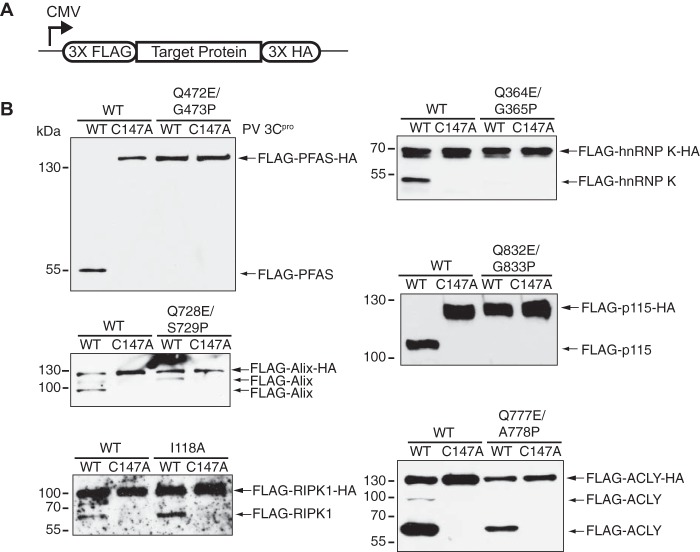
Validation of TAILS-predicted cleavage site by *in vitro* cleavage assay. (A) Schematic of a cytomegalovirus (CMV) promoter-driven mammalian expression construct containing 3×FLAG and 3×HA fused in-frame with the full-length candidate substrate. (B) Lysates from HeLa cells expressing the FLAG-HA-tagged wild-type or mutant candidate substrate were incubated with wild-type or mutant poliovirus 3C^pro^ and immunoblotted for FLAG. N, N-terminal cleavage product.

To biochemically confirm the cleavage sites identified by TAILS, we generated target proteins that contained mutations at the P1 and P1′ positions. We subcloned the wild-type or mutant open reading frame into a mammalian expression vector fused in frame with three copies of a FLAG (3×FLAG) tag and a hemagglutinin (3×HA) tag at the N and C termini, respectively (Fig. 5A). Cell lysates from HeLa cells expressing either wild-type or mutant FLAG-HA constructs were subjected to an *in vitro* cleavage assay using poliovirus 3C^pro^. Incubation with the wild-type but not catalytically inactive (C147A) poliovirus 3C^pro^ resulted in cleavage of wild-type FLAG-HA-tagged PFAS, hnRNP K, ALIX, p115, RIPK1, and ACLY (Fig. 5B). In contrast, mutant versions of these proteins, except for RIPK1 and ACLY, were not cleaved by poliovirus 3C^pro^ at the mutated TAILS-identified cleavage site (Fig. 5B). For FLAG-ACLY-HA, the FLAG antibody detected two cleavage products at ∼63 kDa and ∼90 kDa (Fig. 5B). The larger ∼90-kDa cleavage product, which has the predicted mass of the N-terminal fragment based on the TAILS-identified cleavage site, was not generated from the Gln777Glu/Ala778Pro FLAG-ACLY-HA mutant. The smaller ∼63-kDa cleavage product suggests that poliovirus 3C^pro^ mediates cleavage at an additional site that remains to be determined. Similarly, a second cleavage product of ∼110 kDa, in addition to the TAILS-identified cleavage product of ∼86 kDa, was identified for ALIX using the FLAG antibody. Mutations at the TAILS-identified cleavage site for FLAG-RIPK1-HA failed to block cleavage, generating a single cleavage product of the same molecular mass as the wild-type version. This result suggests that RIPK1 is cleaved at a nearby different site or at multiple sites either directly or indirectly by 3C^pro^.

### TAILS-generated 3C^pro^ candidate substrates are cleaved during virus infection.

**FIG 6:**
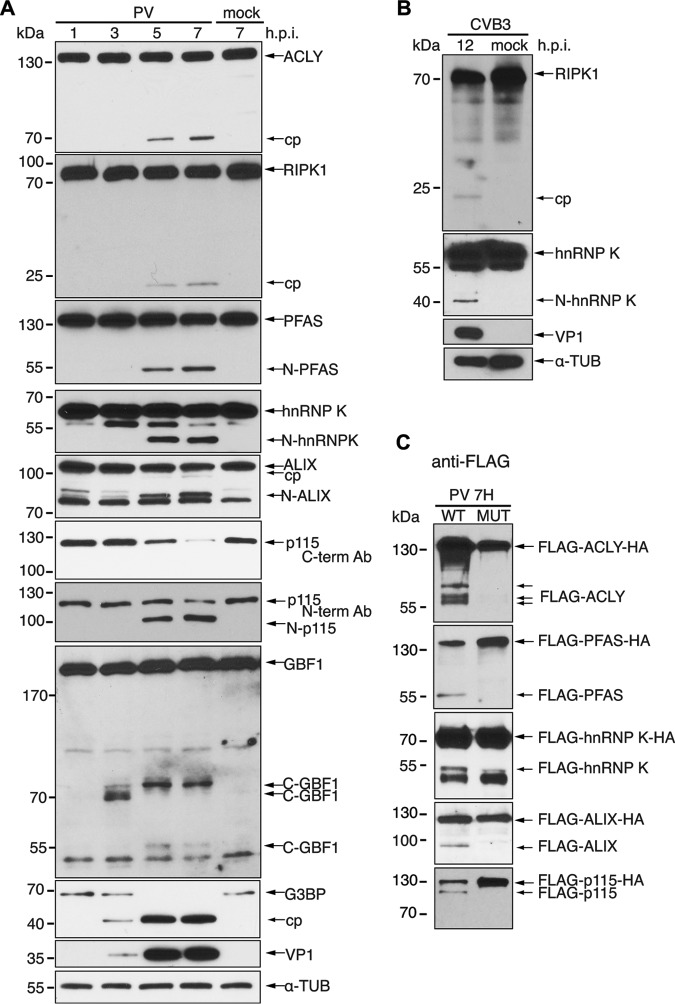
Cleavage of candidate substrates under virus infection. (A) HeLa cells were mock or poliovirus infected (MOI of 10) for the indicated times. (B) HL-1 cells were mock or CVB3 infected (MOI of 50) for 12 h. Candidate substrate, viral structural protein VP1, and α-tubulin were assessed by immunoblotting. (C) HeLa cells transfected with wild-type or mutant FLAG-HA constructs of candidate substrates were mock or poliovirus infected (MOI of 10) for 7 h. Lysates were immunoblotted with FLAG. hpi, hours postinfection; cp, cleavage product; N, N-terminal cleavage product; C, C-terminal cleavage product.

To examine whether cleavage of the target substrates occurs during virus infection, HeLa cells were either mock or poliovirus infected, harvested at different times after infection, and immunoblotted for specific proteins (Fig. 6A). Poliovirus infections were performed at a multiplicity of infection (MOI) of 10 and monitored by detection of VP1 expression. Cleavage of G3BP1, a known substrate of 3C^pro^, was observed starting at 3 h postinfection (hpi), producing the expected cleavage product (Fig. 6A) (41). Similarly, cleavage fragments of ALIX, ACLY, hnRNP K, RIPK1, GBF1, and PFAS were detected by 3 to 5 hpi (Fig. 6A). There was no detectable decrease in the full-length ALIX, ACLY, hnRNP K, RIPK1, GBF1, and PFAS proteins, suggesting that only a subset of these target proteins are cleaved during infection. In contrast, full-length p115 decreased dramatically and was barely detected at 7 hpi. Cleavage fragments of p115 were not detected by this C-terminal antibody, similar to the result observed *in vitro* (Fig. 4 and 6A). However, using the N-terminal p115 antibody, we detected a cleavage product of ∼100 kDa, which is the predicted mass of the N-terminal fragment based on the TAILS results (Fig. 4 and 6A). Thus, the N-terminal cleavage product of p115 is stable during virus infection. Cleavage products of ACLY, PFAS, hnRNP K, and ALIX were of similar molecular masses to the cleavage products observed *in vitro* (Fig. 4). Immunoblotting for RIPK1 detected a smaller protein fragment in infected cells than that detected *in vitro*, consistent with the idea that RIPK1 may have multiple cleavage sites (Fig. 4 and 6A).

We next examined whether the TAILS-identified substrates cleaved during poliovirus infection were also targeted in CVB3-infected HL-1 cardiomyocytes. We previously showed that TDP-43 and hnRNP M are cleaved in CVB3-infected HeLa cells and in cardiomyocytes (44, 45). Using antibodies that cross-reacted with mouse proteins, immunoblotting showed that RIPK1 and hnRNP K were also cleaved in CVB3-infected HL-1 cells at 12 hpi (Fig. 6B). These results confirmed that a subset of TAILS-identified candidate substrates are cleaved in both poliovirus-infected HeLa cells and CVB3-infected HL-1 cells.

We assessed the status of the wild-type and mutant target substrates that were resistant to 3C^pro^ cleavage *in vitro* (Fig. 5B) in virus-infected cells. HeLa cells were transfected with either wild-type or mutant FLAG-HA expression constructs for 48 h, followed by poliovirus infection. Immunoblotting for wild-type FLAG-tagged proteins detected cleavage fragments of ALIX, PFAS, and hnRNP K similar to those observed *in vitro* (Fig. 6C), indicating that the cleaved N-terminal protein fragments are stable and persist during infection. In contrast, several cleavage fragments of FLAG-ACLY were detected that were not observed in the *in vitro* cleavage assay, suggesting that this protein is cleaved at multiple sites during infection, likely by another protease. The full-length FLAG-p115-HA decreased in expression during infection (Fig. 6C), similar to the result observed using the p115 antibody (Fig. 6A); however, we also detected a faint ∼100-kDa band, consistent with observations that the N-terminal cleavage product of p115 is partially stable during infection (Fig. 6C). In contrast to the wild type, FLAG-tagged mutant ACLY, PFAS, hnRNP K, ALIX, and p115 resistant to 3C^pro^ cleavage *in vitro* (Fig. 5) were also resistant to cleavage during infection (Fig. 6C). Thus, these data together with our *in vitro* results shown in Fig. 5 demonstrate that these TAILS-identified proteins are direct targets of 3C^pro^-mediated cleavage during virus infection.

**FIG 7:**
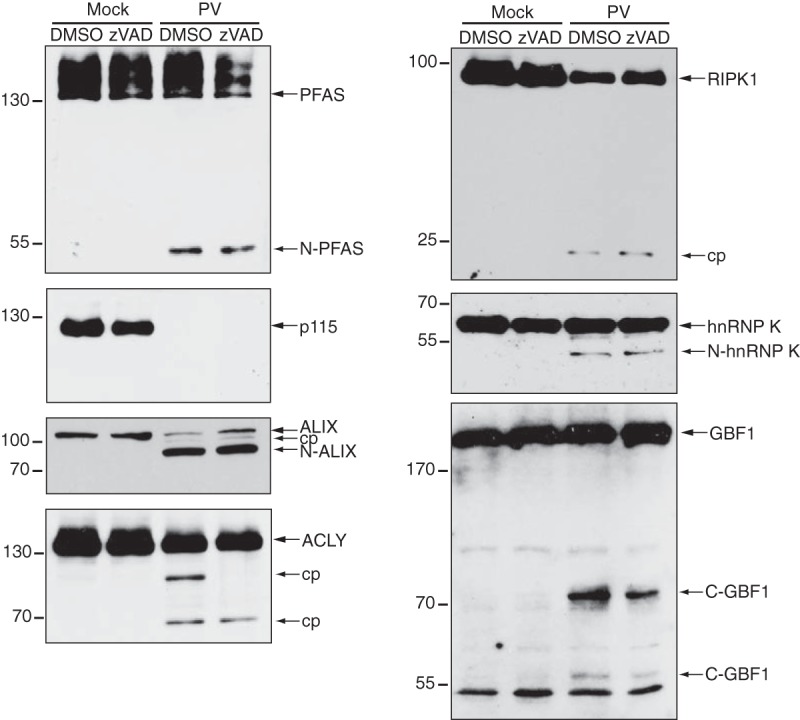
Cleavage of candidate substrates in poliovirus-infected HeLa cells in the presence of zVAD-FMK. HeLa cells were infected with poliovirus (MOI of 10) in the presence of DMSO or 50 μM zVAD-FMK (7 hpi). Candidate proteins were detected by immunoblotting using the indicated antibody. N, N-terminal cleavage product; C, C-terminal cleavage product.

Caspases are activated during enterovirus infections, and poly(ADP-ribose) polymerase (PARP) is cleaved in poliovirus-infected cells (47, 48). PARP cleavage can be prevented by Z-Val-Ala-dl-Asp-fluoromethylketone (zVAD-FMK) treatment in poliovirus-infected cells (45) To assess whether cleavage of the target substrates is a result of caspase activity, we subjected poliovirus-infected cells with zVAD-FMK (Fig. 7). Cleavage or a reduction in full-length protein was still observed for all candidates in poliovirus-infected cells treated with zVAD-FMK. Interestingly, a second cleavage product of ∼90 kDa was observed for ACLY in poliovirus-infected cells treated with dimethyl sulfoxide (DMSO), in addition to the previously observed 65-kDa cleavage product (Fig. 6). Treatment with zVAD-FMK eliminated the 90-kDa cleavage product in poliovirus-infected cells, indicating that a subset of ACLY is targeted by caspases (Fig. 7). Thus, these results further support the idea that the majority of the cleavages on the target proteins identified by TAILS are due to 3C^pro^ activity.

### Depletion of 3C^pro^-targeted substrates affects virus infection.

**FIG 8:**
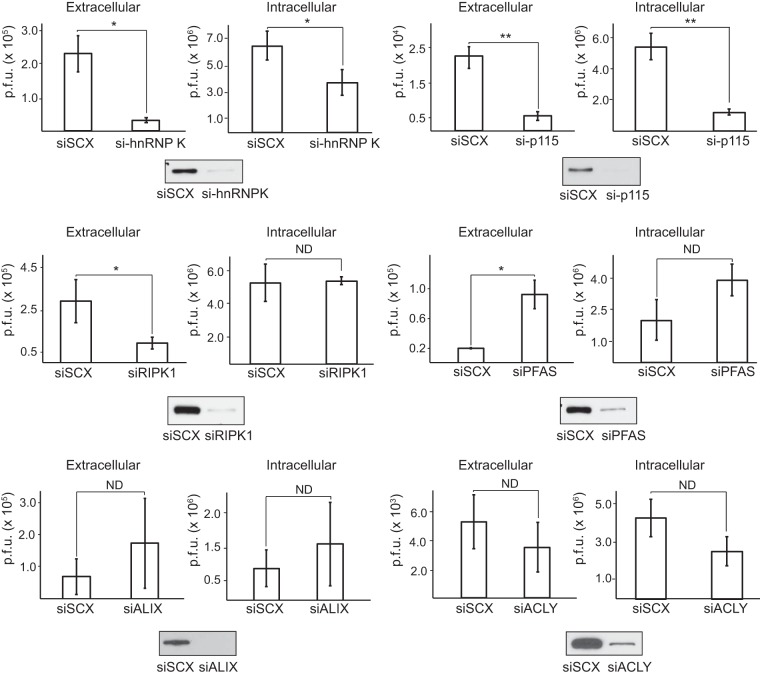
Candidate substrates identified by TAILS modulate poliovirus infection. HeLa cells were transfected with either a scrambled (siSCX) or a candidate-specific siRNA (si- prefix) for 24 to 72 h, followed by poliovirus infection (MOI of 0.1) for 7 h. Titers of extracellular and intracellular virus were determined by plaque assay, and titers were calculated as the number of PFU per milliliter from at least three independent experiments. (*, *P* < 0.05; **, *P* < 0.01). ND, no statistically significant difference. Western blots of the indicated proteins are shown below.

We next explored the biological significance of the target substrates during virus infection using an siRNA knockdown approach. Transfection of HeLa cells with substrate-specific siRNAs for 24 to 72 h led to knockdown of each protein compared to levels in cells transfected with scrambled siRNAs (Fig. 8). Following knockdown, cells were challenged with poliovirus for 7 h, and the medium and cells were subsequently harvested to measure both extracellular and intracellular viral yields by plaque assay. Knockdown of RIPK1, p115, and hnRNP K led to a 3- to 7-fold decrease in extracellular viral yield (Fig. 8). Similarly, knockdown of hnRNP K and p115 resulted in 2- and 5-fold decreases in intracellular viral yield, respectively, indicating a prominent role of these proteins in promoting virus replication. Although extracellular viral yield was decreased by RIPK1 knockdown, intracellular viral yields remained unchanged, indicating a possible role for RIPK1 in viral assembly or release. In contrast, knockdown of PFAS resulted in a 2- and 4-fold increase in intracellular and extracellular virus production, respectively, suggesting that PFAS may be antiviral. Viral yields following either ALIX or ACLY knockdown were not affected. Thus, our *in vitro* TAILS analysis has revealed novel 3C^pro^ substrates that modulate poliovirus infection.

### p115 facilitates poliovirus infection.

**FIG 9:**
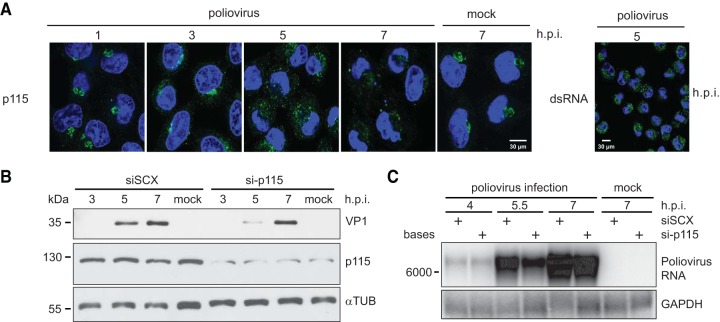
p115 facilitates poliovirus infection. HeLa cells were transfected with either a scrambled (siSCX) or p115 (si-p115) siRNA for 48 h, followed by poliovirus infection (MOI of 1) for the indicated times. (A) Immunofluorescence of endogenous p115 in poliovirus-infected HeLa cells for the times indicated (left). Cells were permeabilized, fixed, and costained for p115 (green; C-terminal antibody) and DNA (blue; Hoechst). An image of HeLa cells stained for viral RNA using an anti-dsRNA antibody at 5 hpi is shown to demonstrate the efficiency of infection at an MOI of 10 (right). (B) Immunoblots of p115, poliovirus structural protein VP1, and α-tubulin are shown. A representative gel is shown from three independent experiments. (C) Northern blot analysis of poliovirus genomic RNA from infected HeLa cells expressing a scrambled (siSCX) or p115 (si-p115) siRNA. GAPDH, glyceraldehyde-3-phosphate dehydrogenase.

p115 is a vesicle membrane-tethering protein that facilitates biogenesis and maintenance of the Golgi apparatus and is involved in regulating the transport of COPI and COPII vesicles between the ER and the *cis*-Golgi network as well as in intra-Golgi transport (49–52). p115 interacts with several Golgi complex-associated proteins, including other tethering proteins, GM130 and giantin, the ARF1 guanine nucleotide exchange factor, GBF1, and the small GTPase, Rab1b, to modulate these functions (53, 54). We showed that p115 is cleaved to near completion during poliovirus infection and that siRNA-mediated knockdown of p115 results in a decrease in viral yield (Fig. 6A and 8), thus revealing a novel proviral factor of poliovirus infection. To assess the biological function of p115 during infection further, we first assessed its subcellular localization during poliovirus infection by immunofluorescence (Fig. 9A). Poliovirus infection disrupts ER-to-Golgi compartment trafficking, resulting in disruption of the Golgi complex and dispersion of Golgi complex-associated proteins, such as GM130 (55). Similarly, in poliovirus-infected cells, p115 began to disperse from a Golgi complex-like staining pattern at 3 hpi. By 5 hpi, p115 was completely redistributed throughout the cytoplasm, forming a multiple punctate pattern (Fig. 9A). At 7 hpi, p115 was barely detected, which correlates with the loss of full-length p115 observed at 7 hpi by immunoblotting using the C-terminal p115 antibody (Fig. 6A). These results are consistent with a previous report that enhanced green fluorescent protein (EGFP)-tagged p115 becomes dispersed in poliovirus-infected HeLa cells (56). Given that dispersion of p115 was detected at 3 hpi, prior to detection of its cleavage at 5 hpi, we suspect that cleavage of p115 occurs after its relocalization from the Golgi compartment (Fig. 6A). Thus, these data confirm that endogenous p115 becomes displaced from the Golgi compartment in poliovirus-infected cells.

We next assessed whether depletion of p115 decreases viral protein production by immunoblot analysis (Fig. 9B). Synthesis of VP1 was monitored over time throughout poliovirus infection in HeLa cells pretreated with either scrambled or p115 siRNA. p115 depletion showed a reproducible delay in VP1 synthesis, most noticeably at 5 hpi. Similarly, loss of p115 decreased poliovirus genomic RNA accumulation by ∼25% at 5.5 and 7 hpi (Fig. 9C), demonstrating that loss of p115 attenuates both viral protein and genomic RNA synthesis. Altogether, these data provide additional evidence to support a facilitative role for p115 in poliovirus replication.

### Cleavage of p115 modulates its association with replication sites.

**FIG 10:**
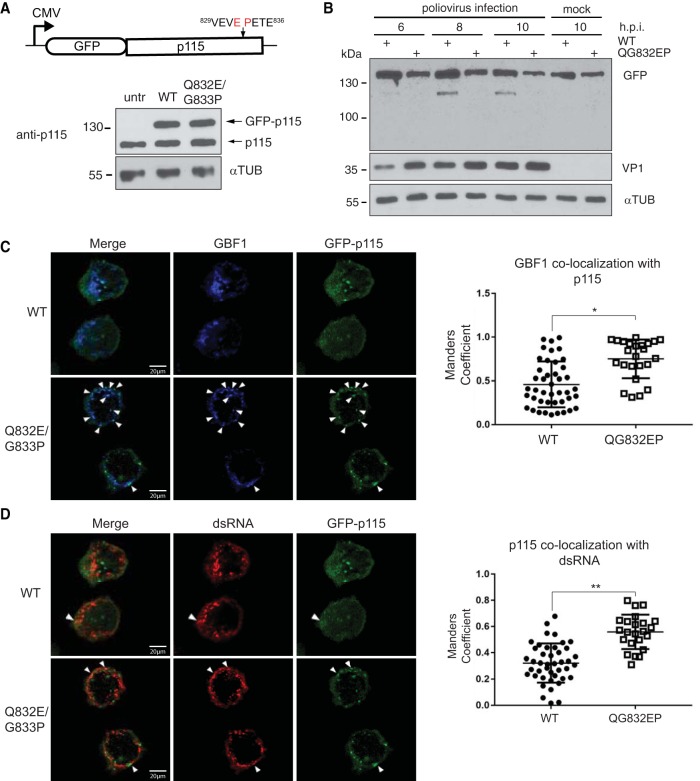
Cleavage of p115 modulates GBF1 association with p115. HeLa cells were transfected with either wild-type (WT) or QG832EP GFP-p115 for 48 h, and expression was assessed by immunoblotting using an anti-p115 antibody in uninfected HeLa cells (A) and following poliovirus infection (MOI of 50) for the indicated times (B). Immunoblots of GFP, VP1, and α-tubulin are shown. untr, untransfected. (C and D) FACS-sorted HeLa cells expressing either wild-type (WT) or QG832EP GFP-p115 were poliovirus infected (MOI of 50) for 8 h. Cells were permeabilized, fixed, and costained with GBF1 (blue) and dsRNA (red) antibodies. Representative images from at least two independent experiments are shown for GBF1 and GFP-p115 (C) and dsRNA and GFP-p115 (D). Dot plot graphs show the Manders' correlation coefficient calculated for the fraction of GBF1 overlapping GFP-p115 (C) and the fraction of GFP-p115 overlapping dsRNA (D) in cells expressing wild-type GFP-p115 (*n* = 46) and QG832EP GFP-p115 (*n* = 25). Shown are quantitations of a representative experiment that showed a reproducible trend from three independent experiments. *, *P* < 0.05; **, *P* < 0.01.

**FIG 11:**
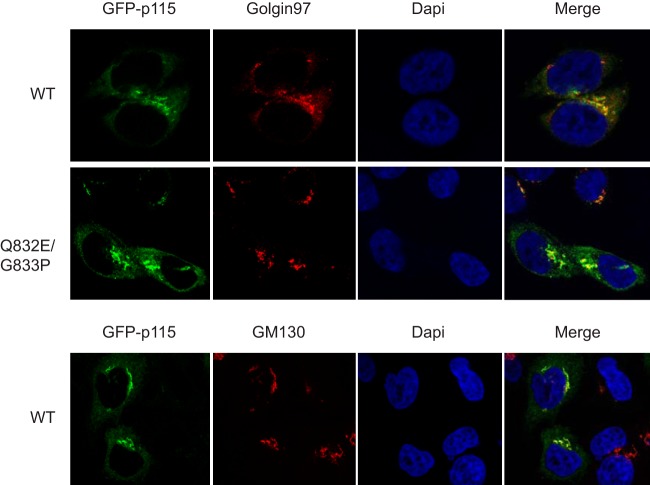
Subcellular localization of wild-type and QG832EP GFP-p115 in HeLa cells. HeLa cells were transfected with either wild-type or QG832EP GFP-p115 for 48 h. Cells were then fixed, permeabilized, and stained for Golgin-97 (red) or GM130 (red), and DNA (blue); 4′,6′-diamidino-2-phenylindole [DAPI].

To determine whether cleavage of p115 is important for infection, we expressed wild-type or cleavage-resistant p115 (QG832EP) in poliovirus-infected HeLa cells. Toward this end, wild-type and mutant forms of p115 were cloned in frame with an N-terminal GFP into a mammalian expression vector (Fig. 10A). Cells were transfected with wild-type and mutant GFP-p115, followed by infection with poliovirus. Immunoblotting of p115 mock-infected cells showed that wild-type and QG832EP GFP-p115 were expressed to levels similar to those of endogenous p115 (Fig. 10A). In poliovirus-infected cells, wild-type but not the mutant QG832EP GFP-p115 was cleaved at 8 and 10 hpi (Fig. 10B). In mock-infected cells, both wild-type and QG832EP GFP-p115 clustered near the nucleus that overlapped the Golgin-97 and/or GM130 signal, consistent with Golgi compartment localization (Fig. 11) (57, 58) and suggesting that the GFP tag does not disrupt p115 subcellular localization.

**FIG 12:**
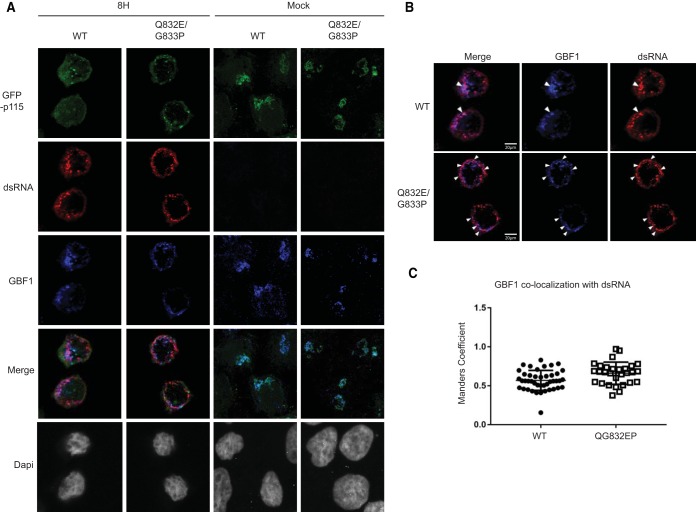
Subcellular localization of wild-type and QG832EP GFP-p115 and GBF1 in HeLa cells during poliovirus infection. (A) FACS-sorted HeLa cells expressing either wild-type or QG832EP GFP-p115 were mock or PV infected (MOI of 50) for 8 h. Cells were permeabilized, fixed, and costained with GBF1 (blue) and dsRNA (red) antibodies. (B) Representative images from at least two independent experiments are shown for the merged images of GBF1 and dsRNA. (C) Dot plot graphs of the Manders' correlation coefficient calculated for the fraction of GBF1 overlapping dsRNA. Shown are quantitations of a representative experiment that showed a reproducible trend from three independent experiments.

Poliovirus replication requires viral 3A protein as well as several cellular proteins, including GBF1 (59). During poliovirus infection, GBF1 transiently colocalizes with double-stranded RNA (dsRNA) and 3A protein, which are markers of replication sites (60–62). dsRNA can be detected by immunofluorescence using a dsRNA antibody (60). Given that p115 interacts with GBF1, we asked whether cleavage of p115 is a prerequisite for GBF1 interaction with dsRNA during poliovirus infection. Fluorescence-activated cell sorting (FACS)-sorted wild-type or QG832EP GFP-p115 cells were infected with poliovirus and subsequently fixed and stained with GBF1 and dsRNA antibodies (Fig. 10C and D). Both wild-type and QG832EP GFP-p115 were dispersed during poliovirus infection, further suggesting that cleavage of p115 is not required for Golgi complex fragmentation (Fig. 12A). We monitored infection at 8 h after virus absorption, a time when wild-type but not QG832EP GFP-p115 is cleaved (Fig. 10B). At 8 hpi, GBF1 signal overlapped the p115 signal in cells expressing wild-type GFP-p115 with an average Manders' correlation coefficient (MCC) of 0.45, indicating that only 45% of GBF1 signal overlapped the wild-type GFP-p115 signal (Fig. 10C). In contrast, in cells expressing QG832EP GFP-p115, the fraction of GBF1 that overlapped the mutant GFP-p115 was reproducibly higher (MCC of 0.74) (Fig. 10C). Similarly, the fraction of p115 that overlapped the dsRNA signal was higher in cells expressing QG832EP GFP-p115 (MCC of 0.59) than in wild-type GFP-p115 cells (MCC of 0.33) (Fig. 10D). The fraction of GBF1 signal that overlapped dsRNA signal in cells expressing wild-type GFP-p115 occurred with a Pearson's coefficient of 0.66 and an average MCC of 0.56, indicating that only 56% of GBF1 signal overlapped dsRNA (Fig. 12B and C). These results are consistent with previous reports that GBF1 associates with dsRNA during poliovirus infection (60). The fraction of GBF1 overlapping dsRNA in cells expressing QG832EP GFP-p115 was similar to that in wild-type GFP-p115-expressing cells (Fig. 12B and C). In summary, these results show that preventing p115 cleavage during poliovirus infection increases the association of the fraction of GBF1 with p115 and the fraction of p115 with dsRNA, thus suggesting that cleavage of p115 may regulate the association of itself and/or GBF1 with replication complexes.

### Cleavage of p115 promotes poliovirus replication.

**FIG 13:**
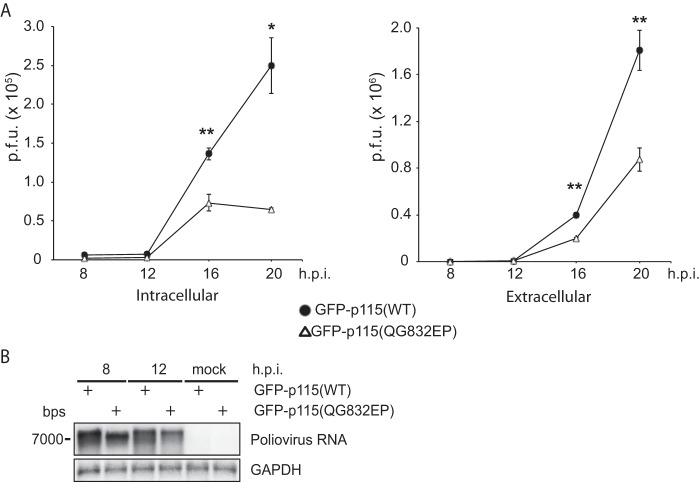
Cleavage of p115 promotes poliovirus infection. (A) Intracellular and extracellular viral titers following poliovirus infection at an MOI of 1 at 8, 12, 16, and 20 hpi of FACS-sorted HeLa cells transfected with wild-type or QG832EP mutant GFP-p115. Average PFU counts ± SD are shown from three independent experiments. **, *P* < 0.001; *, *P* < 0.01. (B) Northern blot analysis of poliovirus genomic RNA in FACS-sorted HeLa cells transfected with wild-type or QG832EP mutant GFP-p115 and infected with poliovirus (MOI of 5).

Given that p115 cleavage may affect replication complexes, we investigated whether cleavage of p115 is required for poliovirus infection. Toward this, FACS-sorted HeLa cells expressing wild-type or QG832EP GFP-p115 were infected with poliovirus at an MOI of 1, and viral titers were monitored at 8, 12, 16, and 20 hpi (Fig. 13A). Expression of the cleavage-resistant GFP-p115 (QG832EP) consistently decreased viral yield compared to the yield with wild-type GFP-p115, with an approximate 4-fold and 2-fold decrease in intracellular and extracellular titers, respectively, observed at 20 hpi (Fig. 13A). Supporting this result, expression of QG832EP GFP-p115 reduced poliovirus RNA synthesis by ∼25% compared to the level in cells expressing wild-type GFP-p115 (Fig. 13B). In summary, these results demonstrate that cleavage of p115 promotes poliovirus infection.

## DISCUSSION

In this, the first comprehensive study using a gel-free proteomics approach that identifies and validates functional host targets of a viral protease, we identified 72 and 34 novel candidate host protein targets of poliovirus and CVB3 3C^pro^, respectively. Knockdown and overexpression validation studies revealed that these target proteins modulate virus infection and so are biologically relevant. For example, cleavage of the vesicle-tethering protein p115 facilitates poliovirus infection and modulates the assembly of host proteins at replication sites. The identification of novel targets of 3C^pro^ adds to the growing list of known substrates that provides a more comprehensive view of the virus-host interactions that contribute to enterovirus infection.

Many substrates, such as PFAS, hnRNP K, and ALIX, generated similar cleavage products *in vitro* and during virus infection, and these cleavage products were stable in virus-infected cells. Although RIPK1 and ACLY were cleaved by 3C^pro^, the masses of the *in vitro* cleavage products were not similar to those produced during infection (Fig. 4 and 6A). We previously reported differences in cleavage product production of hnRNP M during both poliovirus and CVB3 infection compared to production resulting from *in vitro* cleavage (45). Differences between the cleavage pattern *in vitro* and that during infection can manifest for many reasons; e.g., the protein is cleaved by 3C^pro^ at an alternate cleavage site(s) that was not identified for technical reasons by mass spectrometry or is targeted by 2A^pro^ or cellular proteases after the initial and often destabilizing cleavage event. Although we validated cleavage of several 3C^pro^ high-confidence candidate substrates identified by TAILS in virus-infected cells, it was not surprising that a few of the top candidate substrates were cleaved *in vitro* but not during virus infection (e.g., actin, eIF3F, and fascin) (data not shown; see also Table S6 in the supplemental material), indicating that an *in vitro* cleavage assay approach does not fully recapitulate *in vivo* infection, as expected.

TAILS, like all proteomics techniques, can have limitations, hampering its capacity to identify all possible substrates of viral proteases. For example, posttranslational modifications at the N terminus, such as acetylation or cyclization, preclude dimethylation labeling and can reduce the number of quantifiable peptides by mass spectrometry. Other stochastic reasons include duty cycle times of the mass spectrometer that can lead to undersampling, resulting in missed peptides. Moreover, 3C^pro^-derived peptides may also not be of suitable mass and/or achieve sufficient ionization to be identified by mass spectrometry. Indeed, predicted trypsin-digested peptides generated from known 3C^pro^ substrates, such as G3BP1, PABP, and AU-rich element RNA binding protein 1 (AUF1), are either too short or long for efficient ionization and fragmentation to be confidently detected by mass spectrometry (Table S12) (38, 41, 63). Thus, although TAILS identified PTB, a known substrate of poliovirus 3C^pro^, and ∼100 novel candidate protein targets, it is likely that more host targets are yet to be identified through novel alternate proteomic strategies, and this underscores the potential number of impactful host proteins that are targeted by 3C^pro^.

Our two-prong TAILS approach to identify substrates of 3C^pro^ of two enteroviruses was chosen to inventory distinct cleavage specificities and possibly identify common substrates. The IceLogo analysis of the 3C^pro^ cleavage sites suggests that 3C^pro^ is capable of accepting a wider range of amino acids within its active site, therefore allowing for cleavage of a broader range of substrates (Fig. 3) (12). Interestingly, we identified three common proteins (hnRNP K, hnRNP M, and PFAS) as high-confidence candidate substrates of both CVB3 and poliovirus 3C^pro^, which adds to the list of host protein substrates identified as common targets of both 3C^pro^ proteins (45). Whether other host candidate substrates are cleaved among all enterovirus infections warrants further investigation. It is likely that cleavage of a subset of host proteins is strategic whereas other proteins are specific to each enterovirus life cycle, the infected host cell type, and/or its pathogenesis.

Cleavage of some of the validated TAILS-identified candidate targets contributes to virus infection (Fig. 8), emphasizing the utility of TAILS in identifying novel host determinants for enterovirus infection. Both p115 and GBF1, which have been previously characterized during poliovirus infection, are substrates of poliovirus 3C^pro^ during infection (Fig. 4 and 6A). Confirming the central role that p115 plays in viral replication, we found that both knockdown of p115 during virus infection and overexpression of a noncleavable mutant p115 decreased poliovirus titers (Fig. 8 and 13). Both p115 and GBF1 have been associated with a role in enterovirus replication (64). Enterovirus replication occurs on membranous replication organelles that form during infection via a combination of membrane remodeling of Golgi compartment-ER complexes and reorganization of lipid metabolic pathways to generate replication membranes with unique lipid compositions (64, 65). What is the role of p115 cleavage in virus-infected cells? Our results showed that preventing cleavage of p115 promotes GBF1 association with dsRNA (Fig. 12B and C), a marker of replication intermediates. At first, this observation seems counterintuitive as expression of the cleavage-resistant mutant resulted in lower viral yield. Given that a previous report showed that GBF1, which is an essential factor for viral replication, transiently overlaps dsRNA early in infection (60), cleavage of p115 may ensure that the association of GBF1 with dsRNA is transient during virus infection. Consistent with this idea, preventing cleavage of p115 resulted in a greater fraction of GBF1 overlapping cleavage-resistant p115 than in cells that expressed wild-type p115 (Fig. 10C). Given that p115 can interact with GBF1 directly (53), it will be important to determine the affinity of the cleaved p115 and full-length p115 interactions with GBF1. It is worth noting that the presence of the endogenous p115 may mask effects of expression of the cleavage-resistant mutant in infected cells, possibly underestimating effects of cleavage of p115 during infection. One enticing possibility is that stable cleaved fragments (N- or C-terminal) of p115 may have a novel function unrelated to the function of the full-length protein. Indeed, it has been demonstrated that viral protease-mediated cleaved fragments of hnRNP M, G3BP1, and PCBP2 adopt alternate functions that promote virus replication and translation (19, 40, 45, 66). Given that both depletion and blocking cleavage of p115 decrease viral replication and that the N-terminal fragment of p115 is relatively stable during poliovirus infection, cleavage of p115 may lead to a stable cleavage fragment(s) that is subverted during virus infection to support viral replication, possibly through interactions with viral replication complexes.

Another possible function is that p115 may contribute to blocking the secretory pathway during infection. Previous studies have shown that siRNA-mediated depletion of p115, cleavage of p115 by caspases, or expression of its C-terminal caspase cleavage fragment promotes fragmentation of the Golgi apparatus, impairment of secretory trafficking, and induction of apoptosis (67, 68). Cleavage of 3C^pro^ is not through the caspase cleavage site of p115, TEKD_757_, which is relatively close to the 3C^pro^ cleavage site (VEVQ_832_) (Fig. 7). Both GBF1 and p115 become dispersed within the cytoplasm during poliovirus infection (Fig. 10 and 12) (59, 60), indicating that the Golgi apparatus is fragmented in infected cells. Cleavage of p115 by 3C^pro^ along with the expression of poliovirus 3A, 2BC, 2B, and 2C may contribute to Golgi apparatus disassembly and/or block the secretory pathway, thereby suppressing antiviral host cell responses, such as cytokine secretion and antigen presentation (55, 69–72).

TAILS has proven to be effective in identifying substrates of cellular proteases and now for unbiased analysis of viral proteases and the consequences of specific substrates in infection. The TAILS proteomic approach has identified unique and some overlapping substrates for poliovirus and CVB3 3C^pro^, thus paving the way for ongoing studies into the roles of cleavage of the remaining novel host protein targets in enterovirus infection, many of which will likely have specific roles in blocking antiviral responses or in modulating cellular processes to hijack protein function for viral translation, replication, or pathogenesis. Importantly, this unbiased TAILS approach can be applied to identify host cell substrates of other enterovirus proteinases and proteases from other viral families. Identification of host cell substrates of several proteases of different viral families will undoubtedly shed light on the common and various strategies that viruses use to manipulate host cell processes for efficient virus infection.

## MATERIALS AND METHODS

### Protein purification.

His-tagged wild-type and catalytically inactive mutant (Cys147Ala) poliovirus 3C^pro^ proteinases were purified as previously described (73).

### *In vitro* cleavage assay.

*In vitro* cleavage assays were performed as previously described using HeLa or HL-1 cell extracts (73).

### Cell culture and virus stocks.

HeLa cells (already existing collection) were cultured in Dulbecco's modified Eagle medium (DMEM) supplemented with 10% fetal bovine serum (FBS) and 1% penicillin-streptomycin (P-S) at 37°C. Poliovirus (Mahoney type 1 strain; GenBank accession number NC_002058.3) was generated from a pT7pGemPolio poliovirus infectious clone as previously described (generously provided by Kurt Gustin, University of Arizona). HL-1 murine cardiac muscle cells (already existing collection) were cultured in Claycomb medium (Sigma) supplemented with 10% FBS, 1% P-S, 2 mM l-glutamine, and 0.1 mM norepinephrine in ascorbic acid. Poliovirus and CVB3 (Kandolf strain; GenBank accession number M33854.1) were propagated and titers were determined in HeLa cells.

### Virus infections.

Virus was absorbed with HeLa or HL-1 cells at the indicated multiplicity of infection (MOI) (see text and figure legend) for 1 h in serum-free DMEM or Claycomb medium at 37°C, washed by phosphate-buffered saline (PBS), and replaced with complete medium. For virus infections in the presence of Z-Val-Ala-dl-Asp-fluoromethylketone (zVAD-FMK; Calbiochem), zVAD-FMK (final concentration, 50 μM) in serum-free DMEM containing virus was added to Hela cells.

For plaque assays, intracellular and extracellular virus titers were measured from cell supernatant and cell pellet from virus-infected cells, respectively. Cell pellets were prepared by harvesting cells in serum-free DMEM following two washes with PBS and lysing by three freeze-thaw cycles. Plaque assays were prepared as previously described (73).

### Immunoblot analysis.

Equal amounts of protein were resolved by SDS-PAGE and transferred to a polyvinylidene difluoride (PVDF) membrane. Antibodies and dilutions used in this study were as follows: 1:2,000 AIP1/ALIX (Millipore), 1:4,000 RIPK1, 1:7,500 hnRNP K (Santa Cruz), 1:1,000 PFAS (abcam), 1:2,000 ACYL (Millipore), 1:1,000 VDP p115/p115 (Novus Biologicals), 1:2,000 p115 (abcam), 1:1,000 GBF1 (abcam), 1:500 α-tubulin (Santa Cruz), 1:3,000 G3BP1 (BD Transduction Science), 1:3,000 VP1 (Dako), 1:2,000 Flag (Sigma-Aldrich), and 1:1,000 PARP (Santa Cruz).

### Plasmids and transfections.

The full-length open reading frames of the following proteins were PCR amplified and cloned into a p3×Flag-CMV-7.1 (where CMV is cytomegalovirus) vector (Sigma) with a 3×HA tag cloned downstream using XbaI and BamHI sites: hnRNP K (GenBank accession number NM_002149), ALIX (NM_013374), RIPK1 (NM_003804), ACYL (NM_001096), PFAS (NM_012393) and p115 (NM_001290049). Constructs were verified by sequencing. Full-length wild-type and mutant QG832EP p115 proteins were cloned into an mEGFP-C1 vector using XhoI and BamHI sites.

For DNA transfections, HeLa cells were transfected with 1 to 2 μg of plasmid using Lipofectamine 2000 (Invitrogen) according to the manufacturer's protocol. Cells were transfected in antibiotic-free medium for 5 h and then in complete medium for 24 to 48 h. For siRNA transfections, HeLa cells were transfected (30 to 40% confluence) with the following siRNAs using Lipofectamine RNAimax (Invitrogen): hnRNP K (s6739, s6738, and s6737; Ambion), PFAS (s10331, s10332, s10330; Ambion), RIPK1 (s16651; Ambion), and p115 (s16392, s16391, and s16390; Ambion). Knockdown efficiency was validated by Western blotting.

### Immunofluorescence.

HeLa cells on coverslips were fixed with cold 100% methanol for 10 min, washed three times with PBS, and then blocked with 5% bovine serum albumin (BSA) in PBS for 1 h at room temperature or 10% goat serum in PBS overnight at 4°C, followed by 1 h of incubation with primary antibody with 1% BSA or 10% goat serum in PBS at room temperature. The primary antibodies and dilutions used were as follows: 1:100 p115 (Novus Biologicals), 1:400 double-stranded RNA (dsRNA; English & Scientific Consulting Bt), 1:100 GBF1 (abcam), 1:200 Golgin-97 (Cell Signaling), 1:100 GM130 (Cell Signaling), and 1:300 VP1 (Dako). Coverslips were washed three times with PBS and then incubated with 1:500 secondary antibody (goat anti-rabbit or goat anti-mouse Texas Red and goat anti-mouse Alexa Fluor 488; Life Technologies) and 1% BSA or 10% goat serum in PBS and Hoechst to stain for nuclei. Following three washes, coverslips were mounted onto slides using Prolong Gold antifade reagent (Life Technologies). Cells were imaged and analyzed using a Leica SP5 confocal microscope, and pictures were taken using LAS AF software (University of British Columbia [UBC] Life Sciences Institute Imaging Core). For all images, *z*-stacks of approximately 15 slices were captured. Fluorescence intensity of GFP-p115 wild-type- and QG832EP-expressing cells was measured using Fiji software. Colocalization analysis was performed using the JACoP plug-in and calculated for three to five random fields of multiple cells. Pearson's coefficient and Manders' correlation coefficient were determined for wild-type and QG832EP cells expressing GFP at similar fluorescence intensities.

### FACS analysis.

HeLa cells were transfected with either wild-type or mutant QG832EP mEGFP-C1 p115 for 24 h and prepared for fluorescence-activated cell sorting (FACS) analysis by pelleting cells following trypsinization and washing cells in FACS sorting buffer (2 mM EDTA, 2% FBS, 1× PBS) (UBC Life Sciences Institute FACS Core Facility). Cells were filtered, resuspended at 10 to 20 million cells/ml in FACS sorting buffer, and then sorted by FACS using a FACSAria or BD Influx (BD Biosciences) cell sorter. GFP-expressing cells were collected and allowed to recover for 24 h in DMEM supplemented with 20% FBS and 1% P-S.

### Mass spectrometry data analysis.

For samples analyzed on the Orbitrap XL and QExactive, spectra were matched to peptide sequences in the human UniProt protein database (October 2013 release date [http://www.uniprot.org]/) with appended standard laboratory and common contamination proteins using the Andromeda algorithm as implemented in the MaxQuant software package, version 1.3.0.5 (74, 75), using standard settings with light and heavy (13CD2) dimethylation as quantitative labels on lysine and N-terminal residues in the first search and on only lysine residues in the second. In the second search N-terminal modification such as acetylation and pyroglutamation of Gln and Glu residues was used as a variable parameter. Carbamidomethylation (Cys) was used as a fixed modification in both searches, as well as variable methionine oxidation (+15.994915 Da).

Sequence logos were generated with IceLogo with a *P* value of 5% (76). Quadrupole time-of-flight (Q-TOF) data were similarly matched in MaxQuant, version 1.5.2.8, against a UniProt-derived murine proteome database (October 2013 release) with common contaminants added.

The false discovery rate (FDR) was set to 0.01 at the protein level for all searches and to 0.01 or 0.05 at the peptide or peptide-spectrum match (PSM) level, depending on the experiment.

### Statistical analysis.

All statistical analyses were performed using GraphPad Prism. All graphs represent the means ± standard deviations (SD). *P* values were determined using a paired *t* test, and statistical significance was determined as a *P* value of <0.05.

### N-terminal TAILS proteomics.

TAILS was performed as previously described (29, 73). Briefly, equal amounts of HeLa or HL-1 cell lysates were incubated with either wild-type or catalytically inactive Cys147Ala (C147A) mutant purified poliovirus or coxsackievirus 3C^pro^, respectively. Samples analyzed on the linear trap quadrupole (LTQ)-Orbitrap XL were loaded onto a nano-high-performance liquid chromatograph (HPLC) system (Thermo Scientific) coupled to an LTQ-Oribitrap hybrid mass spectrometer (LTQ-Orbitrap XL; Thermo Scientific) through a nanospray ionization source consisting of a fused-silica trap column (length, 2 cm; inner diameter, 100 μm; packed with 5-μm-diameter Aqua C_18_ beads [Phenomenex]), fused-silica fritted analytical column (length, 20 cm; inner diameter, 50 μm; packed with 3-μm-diameter Reprosil-Pur C_18_-AQ beads [Dr. Maisch GmbH]) and a silica gold-coated spray tip (20-μm inner diameter, 6-μm-diameter opening, pulled on a P-2000 laser puller [Sutter Instruments]; coated on an EM SCD005 Super Cool sputtering device [Leica Microsystems]). Buffer A consisted of 0.5% acetic acid, and buffer B consisted of 0.5% acetic acid and 80% acetonitrile (ACN). Gradients were run from 0% B to 15% B over 15 min, from 15% B to 40% B in the next 65 min, then increased to 100% B over a 10-min period, and held at 100% B for 30 min. The LTQ-Orbitrap was set to acquire a full-range scan (*m/z* 350 to 1,800) at a resolution of 60,000 in the Orbitrap and to simultaneously fragment the top five peptide ions in each cycle in the LTQ (minimum intensity, 200 counts). Parent ions were then excluded from MS/MS for the next 180 s. The Orbitrap was continuously recalibrated against protonated (Si(CH_3_)_2_O)_6_ at an *m/z* of 445.120025 using the lock-mass function.

For TAILS using CVB3 3C^pro^, peptides were eluted from stage tips in 80% ACN–0.1% formic acid, SpeedVac concentrated to near dryness, and dissolved in approximately 20 μl of mobile phase A (0.1% formic acid). Peptides were analyzed with an Easy nLC-1000 HPLC system (Thermo Scientific) online coupled to an Impact II high-resolution, high-mass-accuracy quadrupole time of flight (QTOF) system using a CaptiveSpray ion source (Bruker Daltonics) that was modified for minimal postcolumn dead volume as described previously (77). Peptides were loaded onto an in-house packed column (40 cm; inner diameter, 75 μm) packed with C_18_ material (Reprosil-Pur C_18_-AQ beads, 1.9-μm size; Dr. Maisch GmbH) and a silica gold-coated spray tip (20-μm inner diameter, 6-μm-diameter opening, pulled on a P-2000 laser puller [Sutter Instruments]; coated on an EM SCD005 Super Cool sputtering device [Leica Microsystems]).

### Accession number(s).

The MS proteomics data in this paper have been deposited in the ProteomeXchange Consortium (proteomecentral.proteomexchange.org) database under accession number PXD008718.

## Supplementary Material

Supplemental material
