# Supplementary material for: N-Terminomics TAILS Identifies Host Cell Substrates of Poliovirus and Coxsackievirus B3 3C Proteinases That Modulate Virus Infection
Source: J Virol. 2018 Mar 28;92(8):e02211-17. doi: 10.1128/JVI.02211-17 (PMC5874412; doi:10.1128/JVI.02211-17)
Supplement: Supplemental material [file supp_92_8_e02211-17__index.html]

N-Terminomics TAILS Identifies Host Cell Substrates of Poliovirus and Coxsackievirus B3 3C Proteinases That Modulate Virus Infection — Supplemental material 

# N-Terminomics TAILS Identifies Host Cell Substrates of Poliovirus and Coxsackievirus B3 3C Proteinases That Modulate Virus Infection

## Supplemental material

- Supplemental file 1 -

  Table S1–Table S13 (TAILS-identified peptides.)

  XLSX, 1.7M
